# Improvement in duration of erection following phosphodiesterase type 5 inhibitor therapy with vardenafil in men with erectile dysfunction: the ENDURANCE study

**DOI:** 10.1111/j.1742-1241.2008.01947.x

**Published:** 2009-01

**Authors:** M T Rosenberg, P L Adams, T A McBride, J N Roberts, S W McCallum

**Affiliations:** 1 Mid-Michigan Health CentersJackson, MI, USA; 2 GlaxoSmithKlinePhiladelphia, PA, USA; 3 GlaxoSmithKlineOakville, ON, Canada

## Abstract

**Objective:**

The ENDURANCE study evaluated the efficacy of vardenafil, a phosphodiesterase type 5 (PDE5) inhibitor, in men with erectile dysfunction (ED), by measuring the duration of erection leading to successful intercourse using a stopwatch as the assessment instrument.

**Methods:**

This was a randomised, multicentre, double-blind, placebo-controlled, crossover study consisting of a 4-week treatment-free run-in phase after which patients were randomised to either fixed-dose vardenafil 10 mg or placebo (to be administered 60 min prior to intercourse) and entered the first of the two 4-week double-blind treatment periods, separated by a 1-week washout. The primary efficacy end-point was the stopwatch-assessed duration of erection, which was defined as the time from erection perceived hard enough for penetration until withdrawal from the partner’s vagina leading to successful intercourse as measured by Sexual Encounter Profile Question 3 (SEP-3). Secondary efficacy end-points included SEP-2 and SEP-3 success rates, the erectile function domain of the International Index of Erectile Function, global assessment questionnaire, change from baseline in duration of erection and duration of erection not leading to successful intercourse. Safety was assessed by adverse events (AEs), laboratory samples, vital signs and ECGs.

**Results:**

Of the 191 men included in the safety population, 40% had moderate ED and 33% had severe ED at baseline. The duration of erection (least squares mean ± SE) leading to successful intercourse was longer with vardenafil than with placebo (12.81 ± 1.00 min vs. 5.45 ± 1.00 min; p < 0.001). The differences recorded for all secondary end-points were statistically significant in favour of vardenafil compared with placebo (p < 0.001), with the exception of duration of erection not leading to successful intercourse. Vardenafil was well tolerated in this study; the majority of AEs being mild-to-moderate in intensity.

**Conclusion:**

Vardenafil 10-mg therapy provided a statistically superior duration of erection leading to successful intercourse in men with ED compared with placebo.

**What’s known**

The efficacy of phosphodiesterase type 5 (PDE5) inhibitors in restoring erectile function in men with erectile dysfunction (ED) has been evaluated primarily by means of patient-reported outcome measures, including questionnaires and performance scoring.

**What’s new**

This is the first clinical study in which a stopwatch approach was used to measure accurately the duration of erection as it pertains to successful intercourse during PDE5 inhibitor therapy. Results demonstrate that treatment with vardenafil is associated with an increased duration of erection and restoration of erectile function compared with placebo.

## Introduction

Erectile dysfunction (ED) is considered one of the most commonly occurring male sexual disorders (1–3). The high prevalence of ED, as reported in publications on male sexual dysfunction, imposes a significant burden on male health and interpersonal relationships (2,4–9). Approximately 70% of men with ED experience at least one of the following comorbid conditions: hypertension, hyperlipidaemia, diabetes mellitus or depression (7). Because of shared pathophysiological mechanisms, ED is regarded as a sentinel marker of underlying vascular abnormalities (1,10–12). Furthermore, men with ED often do not seek proper medical attention for fear of embarrassment and/or ridicule (13).

In clinical trials, the efficacy of pharmacological agents, including the phosphodiesterase type 5 (PDE5) inhibitors, in the treatment of ED has traditionally been measured by means of patient diaries and questionnaires. These include the Erectile Function domain of the International Index of Erectile Function (IIEF-EF), Sexual Encounter Profile (SEP) Questions, which include SEP 2 (‘Were you able to insert your penis into your partner’s vagina?’) and SEP 3 (‘Did your erection last long enough for you to have successful intercourse?’) and a global assessment question (GAQ; ‘Has the treatment you have been taking over the past 4 weeks improved your erections? Yes/No’). The objective of these studies was to assess the effects of vardenafil and placebo on penetration and maintenance of erection leading to successful sexual intercourse (14–16). Regulatory approval of the PDE5 inhibitors currently available in the USA – vardenafil (17), sildenafil (18) and tadalafil (19) – was based primarily on results obtained with these assessment tools.

Data show that men receiving PDE5 inhibitor therapy have improvement in penile hardness and maintenance of erection, which help them achieve successful intercourse (20,21). However, a stopwatch measurement of the actual duration of erection has not been utilised in a majority of clinical evaluations as an efficacy variable.

ENDURANCE is the first study in which a stopwatch assessment tool was used to measure duration of erection leading to successful intercourse as a primary efficacy end-point following PDE5 inhibitor therapy. The aim was to demonstrate, through the use of this method of assessment, that the PDE5 inhibitor vardenafil improved the duration of erection in men with ED, when compared with a placebo treatment. Stopwatch-assessed duration of erection obtained in this study has also been correlated with the more traditional patient-reported outcome measurements in a separate analysis and found to be well correlated to SEP3 and the EF domain of the IIEF (22).

## Methods

### Study objective

The primary objective of the ENDURANCE study was to determine whether fixed-dose vardenafil 10 mg taken on-demand increased the duration of erection leading to successful intercourse (SEP-3) when compared with placebo in patients with ED.

### Study design

ENDURANCE was a randomised, multicentre, double-blind, placebo-controlled crossover study designed to compare the duration of erection in men with ED receiving fixed-dose vardenafil 10 mg or placebo over a 4-week treatment period. The study consisted of a screening phase, a run-in phase, two treatment phases and a washout period. The protocol, informed consent and other required documents were reviewed and approved by a national, regional or investigational centre independent ethics committee or institutional review board.

Following initial screening, patients entered a 4-week treatment-free, run-in period, during which they were instructed to make at least four attempts at intercourse on four separate days. At least 50% of attempts had to be unsuccessful (see inclusion/exclusion criteria) to qualify for the continuation into the study. Eligible patients from the run-in period were randomised to receive either fixed-dose vardenafil 10 mg or placebo for 4 weeks. This was followed by a 1-week wash-out period, more than sufficient to incur no pharmacokinetic carry over of effect. Patients were then crossed over to receive the alternate study medication from that received during the first 4-week treatment period (i.e. patients who started on vardenafil received placebo and those who started on placebo received vardenafil). Patients were instructed to take the study medication 60 min prior to attempting intercourse. Study visits were scheduled 4 weeks prior to randomisation; on the day of randomisation; and 4, 5 and 9 weeks following randomisation.

### Inclusion and exclusion criteria

The study enrolled men aged 18–64 years who had been in a stable heterosexual relationship for more than 6 months, and who had experienced ED for more than 6 months, according to the National Institutes of Health Consensus Statement (6). In addition, patients had to make at least four attempts at sexual intercourse according to the question in the patient diary (‘Was sexual activity initiated with the intention of intercourse?’) on four separate days, during the untreated run-in period. At least 50% of attempts had to be unsuccessful as determined by the following questions from the patient diary: ‘Were you able to achieve at least some erection (some enlargement of the penis)?’ (SEP-1); ‘Were you able to insert your penis into your partner’s vagina?’ (SEP-2) and ‘Did your erection last long enough for you to have successful intercourse?’ (SEP-3). An unsuccessful attempt was defined by a ‘No’ answer to at least one of these questions. Finally, patients had to register an IIEF-EF domain score of > 5 and < 26 on the second scheduled (randomisation) visit.

The exclusion criteria comprised premature ejaculation (defined as intravaginal ejaculatory latency time < 2 min), penile anatomical abnormalities, primary hypoactive sexual desire, spinal cord injury, retinitis pigmentosa or surgical prostatectomy. Patients with the following conditions were also excluded from the study: severe chronic liver disease; clinically significant chronic haematological conditions; bleeding disorders; significant active peptic ulceration; underlying cardiovascular conditions, including unstable angina pectoris; history of myocardial infarction, stroke, or life-threatening arrhythmia within 6 months of study entry; or resting/symptomatic hypotension. Patients previously unresponsive to PDE5 inhibitor treatments were excluded as well. The following concomitant medications were not allowed: nitrates or nitric oxide donors, anti-androgens, oral or injectable androgens, anticoagulants (except for antiplatelet agents) and alpha blockers. Patients who received any investigational drug (including placebo) within 30 days of screening were ineligible. All patients had to complete written informed consent prior to the study.

### Efficacy assessment

The primary efficacy end-point of this study was the duration of erection leading to successful intercourse as measured by SEP-3. Duration of erection, timed with a stopwatch, was defined as the time from erection perceived hard enough for penetration (start stopwatch) until withdrawal from the partner’s vagina (stop stopwatch). Secondary efficacy end-points included success of insertion as measured by response to SEP-2, success of maintenance of erection as measured by response to SEP-3, erectile function measured by the IIEF-EF domain score and response to the GAQ. Additional secondary efficacy end-points related to the time component of the measuring instrument including duration of erection regardless of SEP-3 response, duration of erection that did not lead to successful intercourse as measured by SEP-3 and the change from baseline in duration of erection. The secondary end-points were not adjusted for multiplicity.

### Safety assessment

Safety and tolerability were evaluated throughout the course of the study by assessing adverse events (AE), clinical chemistry, haematology and urinalysis, 12-lead ECG and vital signs.

### Statistical methods

The intent-to-treat (ITT) population, the population to be used for the efficacy analyses, consisted of patients who were administered at least one dose of study medication and for whom postrandomisation safety data and baseline and postbaseline efficacy data were collected. The safety population, the population for safety summaries, were administered at least one dose of study medication and had postrandomisation safety data collected. According to prestudy sample size calculations, 150 patients were needed to detect a moderate effect size (i.e. ratio of the mean to its SD) of 0.375 with a two-sided significance level of 0.05 and power of at least 90%. Assuming a 20% screen failure rate and a 25% dropout rate, 250 patients had to be screened to randomise 200. The primary efficacy hypothesis was tested at the two-sided 5% significance level to determine if the duration of erection leading to successful intercourse (SEP-3) with vardenafil was superior to that with placebo after 4 weeks of treatment with both therapies. Similar hypotheses were also tested for secondary end-points with no adjustment for multiplicity. The median of the measurements of the primary efficacy variable [duration of erection leading to successful intercourse (SEP-3)] was calculated for each patient then averaged separately for each treatment. Similar medians were also calculated for the secondary efficacy end-points involving duration of erection. The responses for each treatment were compared by a mixed effects model fitting terms for sequence, period, patient within sequence and treatment. The patient was fitted as a random effect. A point estimate and corresponding 95% confidence interval were constructed for the difference between treatments in duration of erection leading to successful intercourse. Similar mixed effects models were also used for the continuous secondary efficacy end-points. The binary end-point GAQ was analysed using a Mainland-Gart test. To be included in an analysis, a patient must have received both treatments.

## Results

### Patient demographics

Of the 346 patients screened for the study, 201 were randomised into the treatment phase, 175 completed the study and 191 were included in the safety and ITT populations. The baseline demographics and ED characteristics of patients in the safety population are displayed in Tables 1 and 2. Patients enrolled in the study had a mean age of approximately 49 years, with a 3.9 years mean duration of ED. Ten per cent of patients used alcohol in moderation, 55% lightly and there was a high incidence of past or present tobacco use (44%, greater than twice the national average) (23) (Table 1). Of the 191 randomised patients included in the safety population, 40% had moderate ED and 33% had severe ED, with a mean baseline IIEF-EF domain score of 13.3 ± 4.6 (Table 2). Baseline comorbidities included hypertension (32%), hypercholesterolaemia (14%), hyperlipidaemia (10%) and diabetes mellitus (7%) (Table 3).

**Table 1:** Baseline demographic data (safety population)

Demographic	All patients, *N*=191
**Age (years)**	
Mean (SD)	49.0 (9.9)
Minimum–maximum	21–64
**Race, *n* (%)**	
White	131 (69)
Hispanic	29 (15)
Black	25 (13)
Asian	5 (3)
Other	1 (< 1)
**Height (cm)**	
Mean (SD)	176.6 (8.0)
Minimum–maximum	152–203
**Weight (kg)**	
Mean (SD)	92.6 (20.5)
Minimum–maximum	57–189
**BMI (kg/m^2^)**	
Mean (SD)	29.6 (6.0)
Minimum–maximum	19–58
**Alcohol use, *n* (%)**	
Abstinent	62 (32)
Light	106 (55)
Moderate	20 (10)
Heavy	3 (2)
**Smoking status, *n* (%)**	
Non-smoker	106 (55)
Smoker, past or present	84 (44)
Passive smoker	1 (< 1)
**Marital status, *n* (%)**	
Married	135 (71)
Never married	35 (18)
Divorced	20 (10)
Widowed	1 (< 1)

BMI, body mass index.

**Table 2:** Baseline ED characteristics (safety population)

Baseline disease characteristic	All patients, *N*=191
**ED aetiology, *n* (%)**	
Organic	92 (48)
Mixed	93 (49)
Psychogenic	6 (3)
**Years since ED first noticed**	
Mean (SD)	4.9 (4.6)
Minimum–maximum	1–30
**Years since ED diagnosis**	
Mean (SD)	3.9 (4.0)
Minimum–maximum	0–23
**ED severity, *n* (%)**	
Total ED (≤ 5)	0
Severe (6–10)	63 (33)
Moderate (11–16)	77 (40)
Mild/moderate (17–21)	43 (23)
Mild (22–25)	8 (4)
**EF domain score**	
Mean (SD)	13.3 (4.6)
Minimum–maximum	6–25

EF, erectile function; ED, erectile dysfunction.

**Table 3:** Summary of select comorbidities and previous PDE5 use at baseline (safety population, *N*=191)

**Comorbidities, *n* (%)**	
Hypertension	61 (32)
Hypercholesterolaemia	26 (14)
Depression	26 (14)
Hyperlipidaemia	20 (10)
Diabetes	13 (7)
**Prior PDE5 inhibitor use, *n* (%)**	
Sildenafil	131 (69)
Tadalafil	37 (19)
Vardenafil	46 (24)

PDE5, phosphodiesterase type 5.

The reasons most frequently cited for premature discontinuation from the study (*n* = 26) were withdrawal of consent (*n* = 10), lost to follow-up (*n* = 9) and non-compliance (*n* = 5). One patient withdrew because of an AE and one patient withdrew because of lack of therapeutic effect of the study medication. Almost three-quarters of patients in the safety population had previously used a PDE5 inhibitor, with sildenafil used most frequently (69%) (Table 3).

### Efficacy

**Figure 1:**
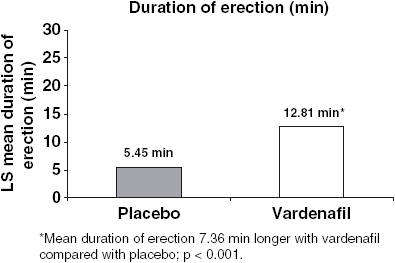
Duration of erection (in minutes) leading to successful intercourse. LS mean duration of erection for vardenafil (12.81 min) and for placebo (5.45 min) over the 4-week treatment period in patients receiving both treatments (*n* = 159), a difference of 7.36 min (95% CI: 5.04–9.67) in favour of vardenafil (p < 0.001)

A total of 191 men were included in the ITT population. During the 4-week treatment period, the least squares (LS) mean duration of erection leading to successful intercourse was statistically superior when patients were treated with vardenafil compared with when patients were treated with placebo (12.81 ± 1.00 min vs. 5.45 ± 1.00 min; p < 0.001; *n* = 159; LS mean ± SE) (Figure 1). This resulted in a difference of 7.36 min between the two treatments (95% confidence interval: 5.04–9.67).

**Figure 2:**
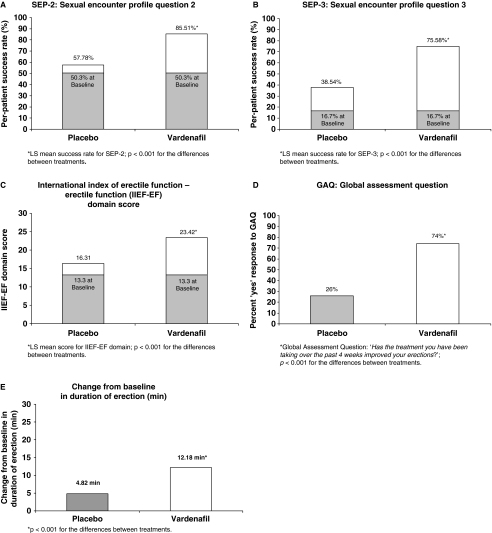
Improvement in erectile function as assessed by SEP-2, SEP-3, IIEF-EF domain score and GAQ in the ITT population, and by change from baseline in duration of erection leading to successful intercourse. Least-square mean scores for individual questions in the patient diary and on the IIEF-EF domain at baseline and after 4 weeks of treatment with either vardenafil 10 mg or placebo. Only men receiving both treatments were included (SEP-2, SEP-3, change from baseline in duration of erection leading to successful intercourse; *n* = 159, IIEF-EF domain and GAQ; *n* = 175). (A) SEP-2 (Were you able to insert your penis into your partner’s vagina?); (B) SEP-3 (Did your erection last long enough for you to have successful intercourse?); (C) IIEF-EF domain scores; (D) Percentage of the ITT population responding ‘yes’ to the GAQ ‘Has the treatment you have been taking over the past 4 weeks improved your erections? (Yes/No)’. The difference between treatments was statistically significant in all cases (p < 0.001). (E) Change from baseline in duration of erection leading to successful intercourse in patients receiving both treatments

Secondary efficacy end-points also demonstrated statistically significant superiority with vardenafil compared with placebo in the same patients, although data were not adjusted for multiple comparisons. Over the 4-week treatment period, the LS mean success rate for SEP-2 was significantly higher when patients received vardenafil compared with when patients received placebo (85.51% vs. 57.78%; p < 0.001; *n* = 159). The difference between the two treatments in LS mean success rate for SEP-2 was 27.73% (95% confidence interval: 21.18–34.28) (Figure 2A). The LS mean success rate for SEP-3 was significantly higher when patients were treated with vardenafil compared with patients treated with placebo (75.58% vs. 38.54%; p < 0.001; *n* = 159) (Figure 2B). The difference in LS mean values was 37.05% (95% confidence interval: 29.03–45.06). Patients also reported statistically significant improvements in erectile function while receiving vardenafil compared with when receiving placebo in terms of the IIEF-EF domain score (23.42 vs. 16.31; p < 0.001; *n* = 175). The difference in LS mean was 7.11 (95% confidence interval: 5.66–8.56) (Figure 2C). In addition, a greater response was reported following vardenafil treatment vs. placebo, as evidenced by the percent of patients with a ‘yes’ response to the GAQ, which asked ‘Has the treatment you have been taking over the past 4 weeks improved your erections?’ (74% vs. 26%; p < 0.001; *n* = 175) (Figure 2D). The duration of erection regardless of SEP-3 response was also significantly greater when patients were receiving vardenafil compared with when they received placebo (13.60 ± 0.99 min vs. 7.59 min ± 0.99; p < 0.001; *n* = 159). The difference in LS mean was 6.01 min (95% confidence interval: 3.8–8.22). When the duration of erection was assessed in attempts where patients did not achieve successful intercourse as measured by response to SEP-3, a statistically non-significant increase was observed when patients received placebo compared with their vardenafil treatment (4.31 ± 0.86 min vs. 3.39 min ± 0.86; p = 0.377; *n* = 59). The difference in LS mean was −0.92 (confidence interval 95%: −3 to 1.15). The change from baseline in duration of erection leading to successful intercourse (for the 159 patients receiving both treatments) was 12.18 ± 0.98 min when treated with vardenafil and 4.82 ± 0.98 min when treated with placebo (LS mean ± SE), a difference of 7.36 min (95% confidence interval: 5.04–9.67; p < 0.001) (Figure 2E).

### Safety

Vardenafil was well tolerated, with the majority of AEs being mild-to-moderate in intensity. The most frequently reported treatment-emergent (began after start of study medication up to 24 h after last dose study medication) AEs (≥ 3%) in patients receiving vardenafil therapy were headache (3%) and flushing (5%). AEs occurring with an incidence of at least 1% are shown in Table 4. One patient reported a serious occurrence of cholecystitis while receiving vardenafil that investigators did not consider drug-related, and another patient reported an occurrence of moderate syncope while receiving vardenafil that resulted in his discontinuation of treatment. This event was considered drug-related, according to the investigator. Vardenafil had no clinically relevant effects on laboratory parameters, vital signs or ECGs.

**Table 4:** Most frequently reported adverse events by patients (≥ 1%) while receiving assigned treatment (safety population, *N*=191)

Adverse event	Placebo, *N*=184, *n* (%)	Vardenafil, *N* = 187, *n* (%)
Any event	20 (11)	32 (17)
Flushing	5 (3)	10 (5)
Headache	4 (2)	5 (3)
Nasal congestion	0	3 (2)
Acute bronchitis	0	2 (1)
Cough	0	2 (1)
Upper respiratory tract infection	3 (2)	1 (< 1)

## Discussion

Traditional methods for measuring the efficacy of PDE5 inhibitors relied on patients’ responses to self-administered questionnaires, including the IIEF-EF domain score, SEP-2, SEP-3 and GAQ, diaries or event logs and interviews (24–26). The main objective of these questionnaires has been to evaluate the effect of PDE5 inhibitors on penetration and maintenance of erection.

Although stopwatch measurements have been previously used to quantify the onset of action of PDE5 inhibitors (27–29), ENDURANCE was the first study in which duration of erection with PDE5 therapy was quantitatively measured as a primary efficacy end-point. A stopwatch approach was used to record accurately in minutes the duration of erection leading to successful intercourse in men with ED. This method thus provided an objective and quantitative measure of the effects of fixed-dose vardenafil 10 mg and placebo, when administered on an as-needed basis 60 min prior to intercourse. Primary efficacy results indicated that patients experienced a greater than two-fold improvement in duration of erection leading to successful intercourse (SEP-3) during a 4-week period when taking vardenafil compared with placebo. Scores for the validated IIEF-EF domain erectile function scale, as well as success rates for SEP-2 and SEP-3, paralleled those obtained with stopwatch measurements. Overall, vardenafil was well tolerated, and the rate and type of AEs reported were consistent with the safety profile of PDE5 inhibitors.

While traditional methods for measuring duration of erection provided some qualitative measures of erectile function in men with ED, the addition of a quantitative measure, such as the stopwatch-assessed duration of erection and its comparison with the currently accepted patient-reported outcome measurements (SEP and IIEF) (22), suggests that it may be a reliable measure of erectile function and could be suitable for use as a primary end-point in future efficacy trials. *In vitro,* vardenafil is a potent and selective PDE5 inhibitor (30). Although *in vitro* affinity does not necessarily translate into potency *in vivo,* the robust effect observed in the ENDURANCE study may in part be attributed to the strong affinity of vardenafil for the PDE5 receptor. This warrants further investigation.

## Conclusion

The efficacy of vardenafil over placebo has been documented many times, but never using a stopwatch to quantify the response in a reproducible objective fashion. In the first PDE5 inhibitor study that assessed duration of erection as a primary efficacy end-point, vardenafil 10 mg produced a statistically superior duration of erection leading to successful intercourse vs. placebo in a general population of men with ED. The results of this study represent an alternative, more objective approach than self-assessment to quantify accurately the efficacy of PDE5 inhibitors in future clinical trials. The stopwatch approach has the opportunity to become the method of choice for efficacy comparisons in the future.
